# Supplementary material for: Time-series transcriptome analysis identified differentially expressed genes in broiler chicken infected with mixed Eimeria species
Source: Front Genet. 2022 Aug 8;13:886781. doi: 10.3389/fgene.2022.886781 (PMC9393255; doi:10.3389/fgene.2022.886781)
Supplement: Supplementary file 2 [file DataSheet1.ZIP › 4dpi_KEGG.Gsea.1625100298584/KEGG_ADIPOCYTOKINE_SIGNALING_PATHWAY.html]

Details for gene set KEGG\_ADIPOCYTOKINE\_SIGNALING\_PATHWAY[GSEA]

|  || Dataset | TMM\_4dpi\_gct\_format\_4dpi\_gct\_format.Class\_4dpi.cls #PC\_versus\_NC.Class\_4dpi.cls #PC\_versus\_NC\_repos |
| Phenotype | Class\_4dpi.cls#PC\_versus\_NC\_repos |
| Upregulated in class | 1 |
| GeneSet | KEGG\_ADIPOCYTOKINE\_SIGNALING\_PATHWAY |
| Enrichment Score (ES) | 0.52750003 |
| Normalized Enrichment Score (NES) | 1.8593911 |
| Nominal p-value | 6.913239E-4 |
| FDR q-value | 0.050194204 |
| FWER p-Value | 0.094 |
Table: GSEA Results Summary

  

Fig 1: Enrichment plot: KEGG\_ADIPOCYTOKINE\_SIGNALING\_PATHWAY      
 Profile of the Running ES Score & Positions of GeneSet Members on the Rank Ordered List

  

| SYMBOL | TITLE | RANK IN GENE LIST | RANK METRIC SCORE | RUNNING ES | CORE ENRICHMENT || 1 | ACSL5 | na | 234 | 1.287 | 0.0401 | Yes |
| 2 | PCK1 | na | 446 | 1.020 | 0.0697 | Yes |
| 3 | CAMKK1 | na | 527 | 0.936 | 0.1064 | Yes |
| 4 | SOCS3 | na | 686 | 0.829 | 0.1316 | Yes |
| 5 | TRADD | na | 711 | 0.810 | 0.1671 | Yes |
| 6 | IRS2 | na | 755 | 0.787 | 0.1999 | Yes |
| 7 | CPT1A | na | 785 | 0.765 | 0.2330 | Yes |
| 8 | CAMKK2 | na | 845 | 0.741 | 0.2624 | Yes |
| 9 | ACSL4 | na | 863 | 0.731 | 0.2948 | Yes |
| 10 | ADIPOR2 | na | 989 | 0.676 | 0.3157 | Yes |
| 11 | NFKBIE | na | 1023 | 0.664 | 0.3437 | Yes |
| 12 | ACSL1 | na | 1172 | 0.612 | 0.3597 | Yes |
| 13 | STK11 | na | 1203 | 0.602 | 0.3851 | Yes |
| 14 | PPARA | na | 1223 | 0.596 | 0.4111 | Yes |
| 15 | CD36 | na | 1326 | 0.568 | 0.4289 | Yes |
| 16 | ACSL3 | na | 1396 | 0.548 | 0.4485 | Yes |
| 17 | PRKAA1 | na | 1419 | 0.542 | 0.4718 | Yes |
| 18 | ACSL6 | na | 1433 | 0.536 | 0.4956 | Yes |
| 19 | STAT3 | na | 1632 | 0.488 | 0.5016 | Yes |
| 20 | JAK2 | na | 1856 | 0.446 | 0.5036 | Yes |
| 21 | RXRA | na | 2286 | 0.377 | 0.4853 | Yes |
| 22 | PTPN11 | na | 2311 | 0.374 | 0.5006 | Yes |
| 23 | AKT3 | na | 2430 | 0.357 | 0.5072 | Yes |
| 24 | ADIPOQ | na | 2680 | 0.326 | 0.5015 | Yes |
| 25 | MAPK9 | na | 2786 | 0.310 | 0.5071 | Yes |
| 26 | IRS4 | na | 2810 | 0.308 | 0.5195 | Yes |
| 27 | NPY | na | 2881 | 0.300 | 0.5275 | Yes |
| 28 | PRKAB1 | na | 3080 | 0.270 | 0.5235 | No |
| 29 | IKBKB | na | 3506 | 0.220 | 0.4981 | No |
| 30 | LEPR | na | 4060 | 0.162 | 0.4594 | No |
| 31 | MTOR | na | 4329 | 0.140 | 0.4434 | No |
| 32 | TRAF2 | na | 4731 | 0.103 | 0.4147 | No |
| 33 | RXRG | na | 4744 | 0.102 | 0.4184 | No |
| 34 | PRKAA2 | na | 4804 | 0.097 | 0.4180 | No |
| 35 | PRKAG2 | na | 5102 | 0.070 | 0.3964 | No |
| 36 | AKT1 | na | 5330 | 0.052 | 0.3798 | No |
| 37 | NFKBIA | na | 5648 | 0.021 | 0.3543 | No |
| 38 | MAPK10 | na | 5730 | 0.014 | 0.3482 | No |
| 39 | RELA | na | 5979 | -0.005 | 0.3277 | No |
| 40 | NFKB1 | na | 6765 | -0.068 | 0.2651 | No |
| 41 | MAPK8 | na | 6831 | -0.073 | 0.2631 | No |
| 42 | TNFRSF1A | na | 7639 | -0.147 | 0.2024 | No |
| 43 | G6PC2 | na | 8058 | -0.185 | 0.1761 | No |
| 44 | PRKAG1 | na | 8326 | -0.211 | 0.1635 | No |
| 45 | NFKBIB | na | 8760 | -0.257 | 0.1392 | No |
| 46 | SLC2A1 | na | 9227 | -0.312 | 0.1147 | No |
| 47 | PRKAB2 | na | 9322 | -0.325 | 0.1219 | No |
| 48 | CHUK | na | 9400 | -0.336 | 0.1311 | No |
| 49 | ADIPOR1 | na | 9523 | -0.352 | 0.1372 | No |
| 50 | TNFRSF1B | na | 10094 | -0.428 | 0.1093 | No |
| 51 | PCK2 | na | 10229 | -0.450 | 0.1190 | No |
| 52 | PPARGC1A | na | 11134 | -0.645 | 0.0733 | No |
Table: GSEA details [plain text format]

  

Fig 2: KEGG\_ADIPOCYTOKINE\_SIGNALING\_PATHWAY      
 Blue-Pink O' Gram in the Space of the Analyzed GeneSet

  

Fig 3: KEGG\_ADIPOCYTOKINE\_SIGNALING\_PATHWAY: Random ES distribution      
 Gene set null distribution of ES for **KEGG\_ADIPOCYTOKINE\_SIGNALING\_PATHWAY**
